# Structural basis of antifreeze activity of a bacterial multi-domain antifreeze protein

**DOI:** 10.1371/journal.pone.0187169

**Published:** 2017-11-06

**Authors:** Chen Wang, Svetlana Pakhomova, Marcia E. Newcomer, Brent C. Christner, Bing-Hao Luo

**Affiliations:** 1 Department of Biological Sciences, Louisiana State University, Baton Rouge, Louisiana, United States of America; 2 Department of Microbiology and Cell Science, Biodiversity Institute, University of Florida, Gainesville, Florida, United States of America; Russian Academy of Medical Sciences, RUSSIAN FEDERATION

## Abstract

Antifreeze proteins (AFPs) enhance the survival of organisms inhabiting cold environments by affecting the formation and/or structure of ice. We report the crystal structure of the first multi-domain AFP that has been characterized. The two ice binding domains are structurally similar. Each consists of an irregular β-helix with a triangular cross-section and a long α-helix that runs parallel on one side of the β-helix. Both domains are stabilized by hydrophobic interactions. A flat plane on the same face of each domain’s β-helix was identified as the ice binding site. Mutating any of the smaller residues on the ice binding site to bulkier ones decreased the antifreeze activity. The bulky side chain of Leu174 in domain A sterically hinders the binding of water molecules to the protein backbone, partially explaining why antifreeze activity by domain A is inferior to that of domain B. Our data provide a molecular basis for understanding differences in antifreeze activity between the two domains of this protein and general insight on how structural differences in the ice-binding sites affect the activity of AFPs.

## Introduction

Ice binding proteins (IBPs) are characterized by their ability to specifically bind to one or multiple planes of ice crystals [1]. Antifreeze proteins (AFPs) are a class of IBPs that have been documented in a number of cold-tolerant fish [2, 3], insect [4], bacterial [5, 6], fungal [7], and plant [8] species, and this phenotype permits them to prevent and/or control ice crystal formation. When bound to the ice surface, AFPs depress the freezing point without significantly altering the melting point [9]. The difference between the freezing and melting point, referred to as the thermal hysteresis (TH) gap, is often used as an indicator of AFP activity [10]. It is thought that TH is caused by the Kelvin effect because AFP binding to the ice surface generates a micro-convex structure that is thermodynamically less favorable for water molecules to bind compared with a flat ice surface [11, 12]. At subzero temperatures, small ice crystals recrystallize into larger ones to minimize the surface energy (i.e., Ostwald ripening). Importantly, ice recrystallization damages cell membranes, and therefore is one of the most lethal stresses a cell encounters under frozen conditions [13]. AFPs significantly inhibit this process after binding to ice (RI, recrystallization inhibition) [14, 15], either by preventing water molecules from leaving the ice crystals or acting as a surfactant to reduce the surface tension. Since this activity conserves the boundaries among ice grains, AFPs are hypothesized to enhance microbial survival in ice matrices, such as those found in deep Antarctic glacial ice [5, 16, 17].

While all AFPs share the similar function of ice binding, their sequences and structures differ widely, making it difficult to infer their molecular detail responsible for this property. The AFPs of Antarctic fish were the first to be discovered [2] and have been studied extensively. Based on their structural features, four types of fish AFPs are recognized [18]. The type I fish AFPs have the simplest structures and may consist of a single Ala rich α-helix [19]. Recently, Sun et al. reported the crystal structure of an isoform of type I fish AFP isoform, Maxi, which consists of a four helical bundle that retain 400 water molecules inside its core [20]. Type II and type III fish AFPs are both relatively small globular proteins. Type II fish AFPs are stabilized by disulfide bonds [21], while type III fish AFPs are held together mainly through a hydrophobic core [22]. There is currently no structure of type IV fish AFPs reported. Most of the structurally characterized AFPs adopt a β-solenoid / helical structure with various cross sections [23], contain repeating motifs, and have well aligned side chains on their ice binding sites [24–29]. However, there are several β-helical AFPs that deviate from this structural regularity and conservation [30–34]. In general, AFPs form three-dimensional structures maintained by hydrogen bonds, electrostatic interactions and disulfide linkage, but the traditional hydrophobic core is sometimes not present [20, 23, 35, 36]. Since AFPs are synthesized, folded and function at low temperature, a stabilized structure is probably not essential.

The ice-binding site (IBS) is the functional region of an AFP. Due to low sequence similarity between AFPs, there are almost no common sequences or structural folds to aid in identifying the IBS. Therefore, the most direct way to determine the IBS of an AFP is using site-directed mutagenesis to systematically study how changes in the property and/or size of residues affects antifreeze activity. Although the overall structures of AFPs vary significantly, it has been found that the IBSs do share common features. IBSs are characterized by a large, relatively flat and hydrophobic plane on the protein surface, and commonly have repeating motifs [23]. It remains elusive how AFPs bind rapidly and irreversibly to ice, outcompeting 55 M of liquid water. Early work on type I fish AFPs revealed a regular array of threonines on the protein surface, suggesting hydrogen bonding might be important for binding to ice [37, 38], but mutagenesis studies of type I and type III fish AFPs implied a role for hydrophobic residues in the flat-binding surface [39–41]. It was later proposed that ordered waters on the IBS might be released into the bulk solvent on AFPs binding to ice, with a gain in entropy driving the process in the direction of binding [42]. Recent modelling studies proposed that AFPs might organize water molecules into an ice-like structure that resembles the quasi-liquid layer of water next to the ice surface around the hydrophobic groups [43–45]. It has even been suggested the AFPs bring their own “ice” to the ice surface [25, 29, 46], and recent crystal structures of several AFPs with constrained ice-like waters on the hydrophobic IBS strongly support this hypothesis [20, 24]. However, it remains unknown whether this putative binding mechanism is common to other AFPs.

Previously we examined the expression, ice binding affinity, and effect on cell viability during freeze-thaw cycling of a bacterial protein [5, 47, 48]. This 54 kDa AFP (IBPv) was secreted by a bacterium within the family *Flavobacteriaceae* (strain 3519–10) that was isolated from a depth of ~3.5 km in the Vostok Ice Core [5]. Based on the recombinant IBPv exhibiting TH of 2.2°C at a concentration of 53μM, it is categorized as a hyperactive AFP [47]. According to primary sequence, IBPv was predicted to consist of two separate ice binding domains. Although domain B is superior in interacting with ice, the addition of domain A enhances the TH, indicating collaborative effects between the domains during the ice binding process [47]. Here we report the first high resolution crystal structure of the multi-domain AFP, IBPv. Site-directed mutagenesis was used to identify the putative ice binding sites of each domain based on structural and functional analyses. These data provide a molecular basis for understanding differences in antifreeze activity between the two domains of IBPv and general insight on how structural differences in the IBS affect the TH activity of AFPs.

## Materials and methods

### Expression of IBPv and its mutants

Recombinant IBPv was purified as previously described [47]. The IBPv encoding sequence, with the signal peptide removed and 6 × His tag at the C-terminus, was inserted into a pET-21a vector. The plasmid was transformed into BL21 cells. An overnight culture grown in LB medium containing 100 μg/ml of ampicillin was diluted 1:100 (v/v) into fresh LB medium and incubated at 37°C until an O.D. of 0.8. Protein expression was then induced by addition of 1 mM IPTG. Cells were harvested after incubating for 18h and then frozen at -80°C. The pellet was resuspended in TBS buffer (20 mM Tris, 150mM NaCl, pH 7.5) that contained 10μg/mL DNase I and 100μg/mL lysosome. Cells were lysed through sonication. After centrifugation, the supernatant was applied to a Ni-NTA agarose (Qiagen) column and further purified through a gel filtration column (Superdex 75; GE AKTA purifier). The purified protein was concentrated with a centrifugal filter device (Millipore, 10kD cut off) to reach a concentration of 50mg/mL.

Site-directed mutagenesis was conducted with the Quikchange mutagenesis kit (Agilent Genomics). Primers were designed to overlap with at least 10bp upstream and downstream of the mutation site. The PCR product was treated with DpnI to remove the template and then transformed into DH5α cells. Plasmids were extracted and mutations were confirmed with DNA sequencing. IBPv mutants were purified using similar procedure as wild type.

### Protein crystallization

Crystals of the enzyme were obtained using the hanging drop vapor-diffusion method by mixing equal volumes of protein (50 mg/mL concentration in TBS buffer) and the reservoir solution (15–20% PEG 3350, 0.2 M ammonium nitrate) at 22°C. The crystals grew in approximately one month and belonged to the rhombohedral space group *R32* with *a* = 119.83 Å, *c* = 367.76 Å.

### Data collection

Prior to the data collection, a suitable crystal was dipped for several seconds in a modified mother liquor solution with the addition of 15% glycerol as a cryoprotectant. Diffraction data were collected at 100 K at beamline X6A in the National Synchrotron Light Source of the Brookhaven National Laboratory at a 1.00 Å wavelength. The images were processed and scaled using the HKL2000 program suit [49]. Data collection and data processing statistics are given in Table 1.

**Table 1:** Data collection and refinement statistics.

**t**	
Space group	*R32*
Cell dimensions	
*a*, *c* (Å)	119.83,367.76
Resolution (Å)	40–1.75 (1.81–1.75)*
*R*_sym_	0.091 (0.732)
*I* / σ*I*	29.1 (3.2)
Completeness (%)	99.9 (100)
Redundancy	8.6 (8.5)
**Refinement**	
Resolution (Å)	36.08–1.75
No. reflections	99,891
*R*_work_ / *R*_free_	16.85/19.05
No. atoms	
Protein	6,039
Ligand/ion	4
Water	684
*B*-factors	
Protein	28.1
Water	31.5
R.m.s. deviations	
Bond lengths (Å)	0.012
Bond angles (°)	1.457

*Values in parentheses are for the highest-resolution shell.

### Crystal structure determination

IBPv shares low sequence identity (29%) with the ice binding protein *Le*IBP from Arctic yeast *Leucosporidium* sp. for which crystal structure is known [31]. According to the sequence alignment, there are two copies of *Le*IBP per one molecule of IBPv. The molecular replacement procedure was applied to locate a solution using the program MOLREP [50]. A monomer of *Le*IBP (PDB accession code 3UYU) was used as a search model. A total of four *Le*IBP monomers were located in the asymmetric unit of IBP.

The positioned MR model was refined using the maximum likelihood refinement in REFMAC [50] with the TLS parameters generated by the TLSMD server [51]. TLS tensors were analyzed, and anisotropic B-factors were derived with TLSANL program [52]. The program Coot was used for model building throughout the refinement [53]. The final model consists of protein residues 23–445 for protein molecule A, 23–446 for protein molecule B, one nitrate anion and 684 water molecules. Alternate conformations have been built for protein residues T127, R221, I247, V345, I413 (molecule A) and T127, T179, K184, R221 (molecule B). 98% of residues lie in the favored region of the Ramachandran plot with no residues in the disallowed region. The atomic coordinates and structure factors (code 5UYT) have been deposited in the Protein Data Bank.

### Circular dichroism spectroscopy

The CD spectra of the IBPv, IBPv_a and their mutants were measured from 200nm to 250nm with a Jasco Model J-815 circular dichroism spectrometer. The protein solutions were diluted in TBS to around 0.5mg/mL and placed in a 0.1cm path length cell. Data were collected at 0.5nm bandwidth and the scan speed was 50nm/min. Three scan values were averaged. CD data is available in S2 Table.

### Thermal hysteresis assay

TH activities of the IBPv, IBPv_a and their mutants were measured with a nanoliter osmometer (Otago Osmometer, Dunedin, New Zealand) connected to a Leica DM LB2 microscope. First, cargille B emersion oil was applied to the sample holes. Then about 20nL of sample was loaded into the oil with a capillary tube connected to a syringe. The temperature was rapidly decreased to below -20°C to initiate freezing process. After ice formation, the temperature was gradually raised until a single ice crystal (diameter in 1–10 μm range) remained in the droplet. The temperature was recorded as the melting temperature. The sample was then slowly cooled until a rapid burst of ice crystal growth was observed. The temperature was recorded as the freezing temperature. The difference between the freezing and melting temperatures was calculated as the TH. TH data is available in S1 Table.

### Structural alignment of several β helical AFPs

The structural alignment of IBPv_a, IBPv_b and their homologous proteins, namely *Le*IBP (PDB: 3UYU) [31], *Tis*AFP6 (PDB: 3VN3) [30], *Tis*AFP8 (PDB: 5B5H) [34], *Ff*IBP (PDB: 4NU2) [33] and *Col*AFP (PDB: 3WP9) [32], was performed using Multiseq under VMD platform [54]. The structures were colored by structural similarities (Qres) or sequence similarities (BLOSUM 60). All the structure figures were generated using Pymol [55].

## Results

### Overall structure of IBPv

**Fig 1:**
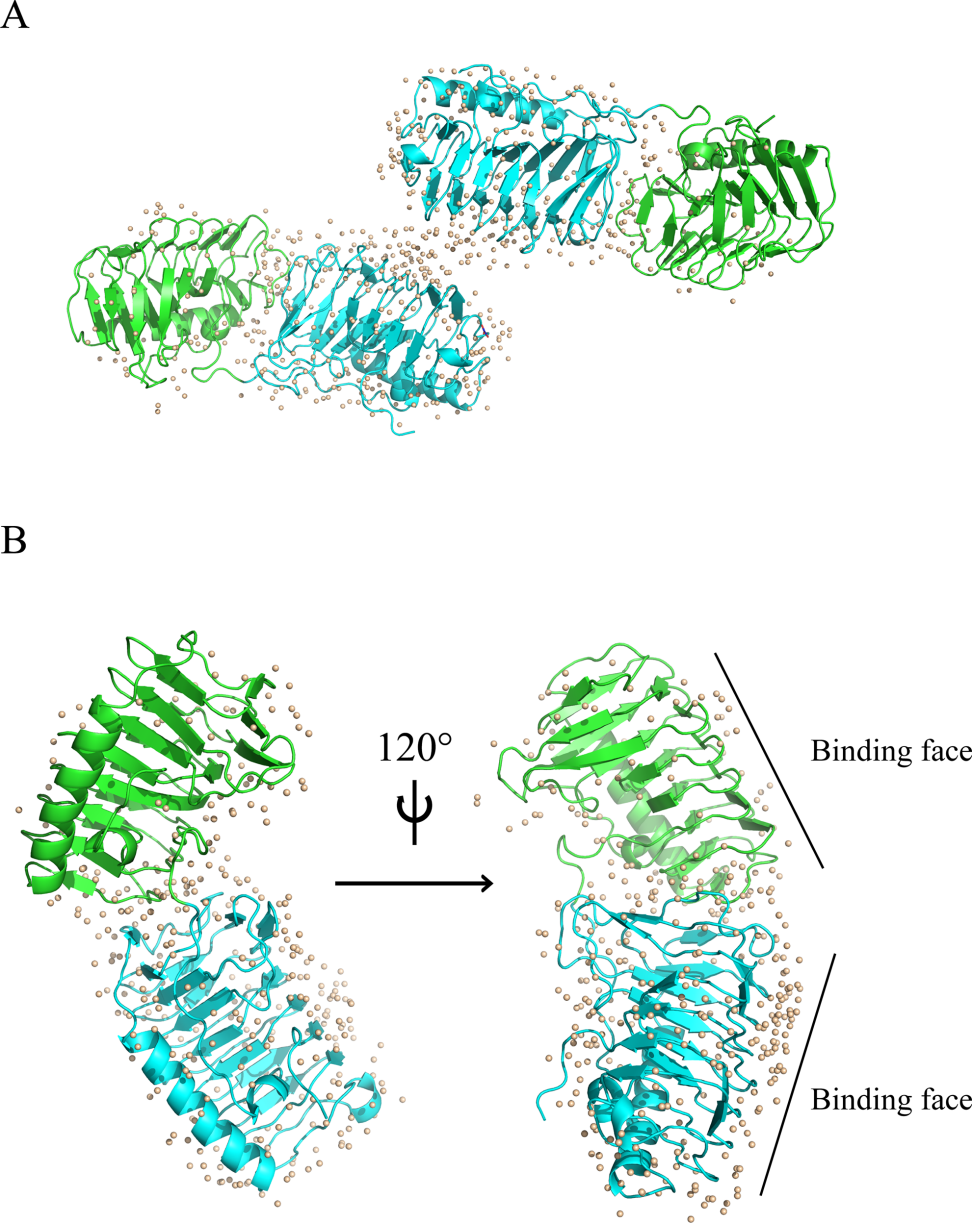
The overall structure of IBPv. (A) Two protein molecules were observed in the asymmetric unit of the IBPv crystal. Each protein molecule contains two ice binding domains, which are colored green and cyan, respectively. Water molecules are depicted as gray spheres. (B) The overall structure of a single IBPv molecule. More water molecules (gray spheres) were bound with B (cyan) domain than A (green) domain.

**Fig 2:**
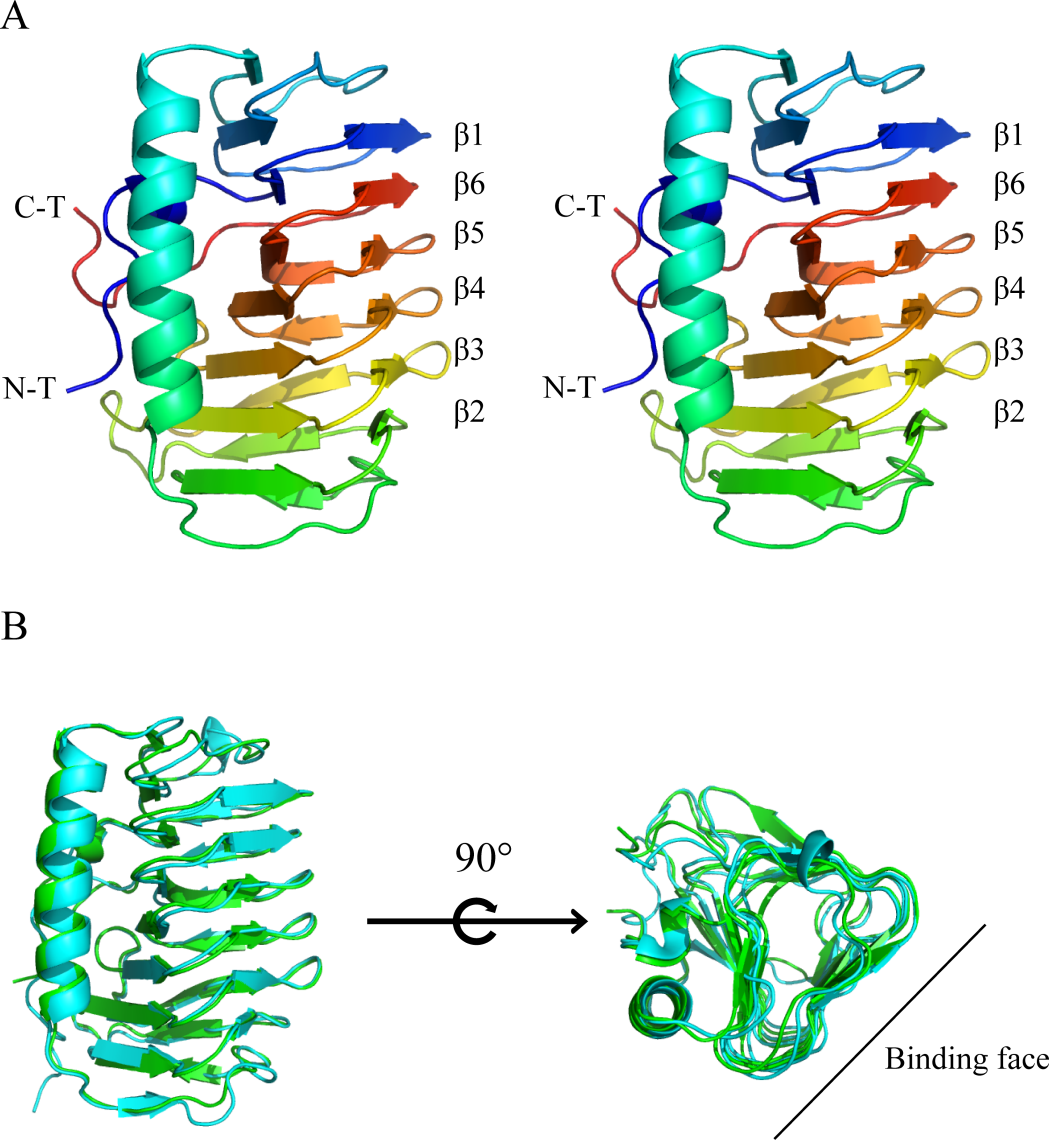
Comparison of two domains of IBPv. (A) The stereo view of the secondary structure of domain A, colored from blue (N terminal end) to red (C terminal end). (B) Structural alignment of domain A (green) and domain B (cyan).

The 1.75Å IBPv structure was determined by molecular replacement and the overall structure is shown in Fig 1. Data collection and refinement statistics are summarized in Table 1. Two similar domains were identified in each protein molecule. Domain A and B is comprised of residues 23 to 229 and 230 to 445, respectively, in the primary sequence. Each domain contains a six-loop right-handed β-helix (solenoid) with a triangular cross section, and an α-helix, which runs parallel on one side of the β-helix (Fig 1B). The β-helix solenoid is arranged in an order of β1-β6-β5-β4-β3-β2 (Fig 2A). The structures of the two domains were aligned and superimposed well with RMSD of 0.68 Å on the protein backbone (Fig 2B). However, no electron density was observed for the last 88 amino acids of the C-terminus. Previously we expressed the truncated protein without the C-terminal 88 residues and showed that it possessed a TH ~0.5°C lower than that the full length protein at concentrations above 20μM [47]. However, the contribution of the C-terminus to TH activity remains unexplained.

Ordered water molecules are located around both domains; however, more water molecules are bound to domain B than domain A (Fig 1B). Previous studies have shown that domain B had higher TH activity and was superior at binding to ice compared to domain A [47]. These results suggest that the number of ordered water molecules may correlate with higher antifreeze activities.

### IBPv is stabilized by hydrophobic interactions and disulfide bonds

**Fig 3:**
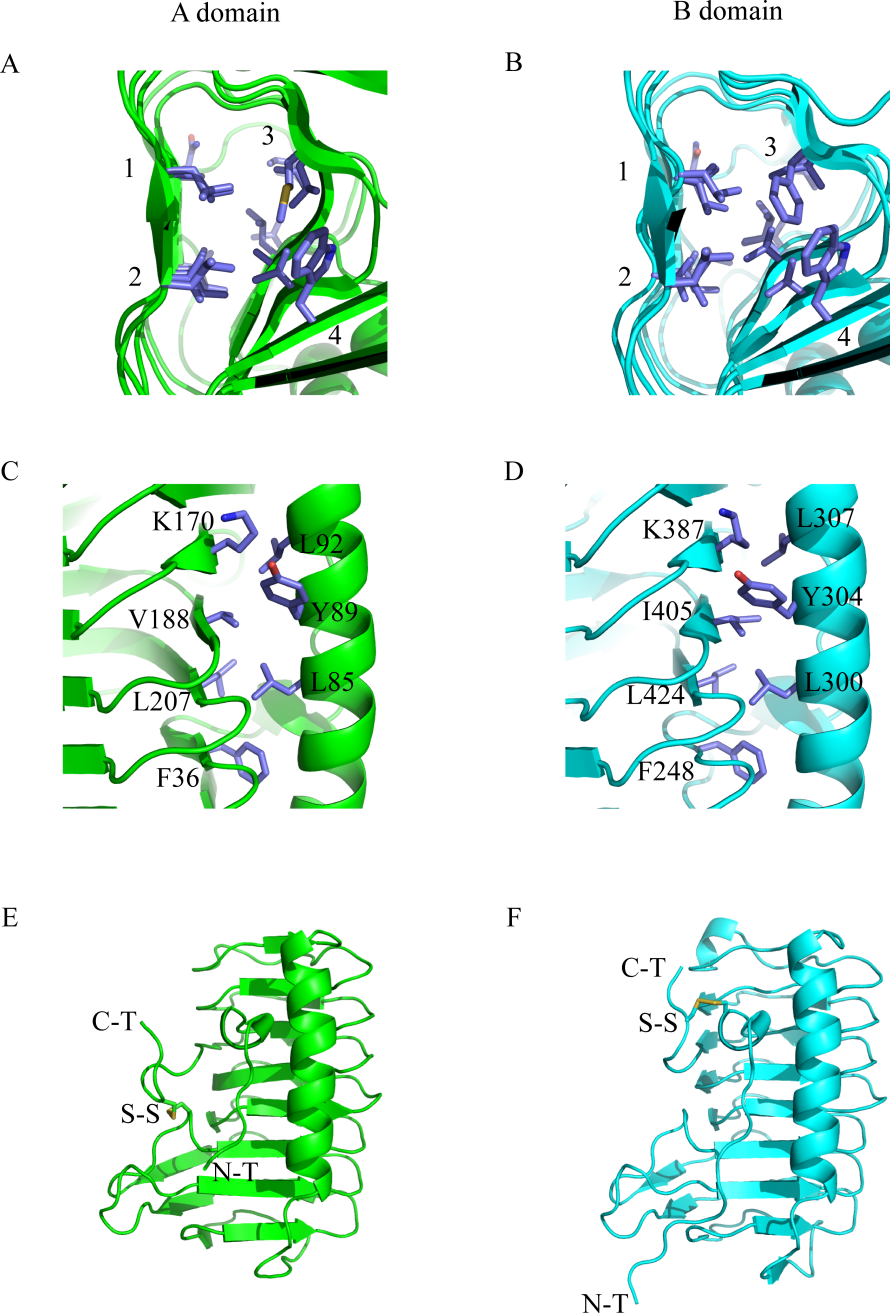
Hydrophobic interactions and disulfide bonds. (A) & (B) The aligned hydrophobic residues inside the β-helix of domain A and B. In each core, four rows of tightly packed side chains were found, those residues were summarized in Table 2. (C) & (D) The hydrophobic interactions between the α-helix and β-helix for domain A and domain B. The amino groups of K170 and K387 and the hydroxyl groups of Y89 and Y304 point outward, allowing the hydrocarbon chains and the phenyl groups to from hydrophobic interactions with other non-polar side chains at the interfaces. (E) & (F) The intra-domain disulfide bond formed in A domain and B domain. N-T stands for N terminal end, C-T stands for C terminal end, and S-S stands for disulfide bond.

The IBPv structure suggests that hydrophobic interactions play an important role in protein stability, consistent with our previous observation that IBPv was quite stable at higher temperatures, with a Tm of 53.5°C [47]. As illustrated in Fig 3A and 3B, four rows of well aligned and tightly packed side chains exist inside of the β helix for each domain. Detailed information on the position and composition of residues are summarized in Table 2. Of the 38 residues inside of the β helix, 35 are hydrophobic and only 3 (N44 in domain A, and N256 and T432 in domain B) are hydrophilic. However, the polar side chains of the hydrophilic residues point away from the hydrophobic core. In addition, the N44 side chain is hydrogen bonded with S48, while N256 with T432 and T260, making no unpaired polar group interfering with the hydrophobic core. Furthermore, hydrophobic residues were also found at the interface between the β-helix and the α-helix in each domain as shown in Fig 3C and 3D. Although residues containing polar groups were found at the interface for both domains (K170, Y89 on A domain and K387, Y304 on B domain), the amino and hydroxyl groups point outward towards the solvent.

**Table 2:** Summary of hydrophobic core of each domain.

	Row	1	2	3	4
Domain A	Outside	L177	M183	L148	W169
	↓	L196	L202	V175	V187
		V215	V220	I194	A206
		N44	I50	I213	L35
	Inside		V67	V42	V54
Domain B	Outside	L394	F400	L365	W386
	↓	I413	I419	V392	V404
		T432	A437	V411	V423
		N256	V262	I430	I247
	Inside		V281	L254	V266

Each domain contains one intra-domain disulfide bond, namely C165-C226 in domain A and C244-C443 in domain B (Fig 3E & 3F). C226 and C443 are located on the C terminals while C165 and C244 are located on the loop region of the β-helices. These two disulfide bonds keep the C-terminal ends close to the cores and may prevent the protein from denaturing.

### Determination of the ice binding sites

**Fig 4:**
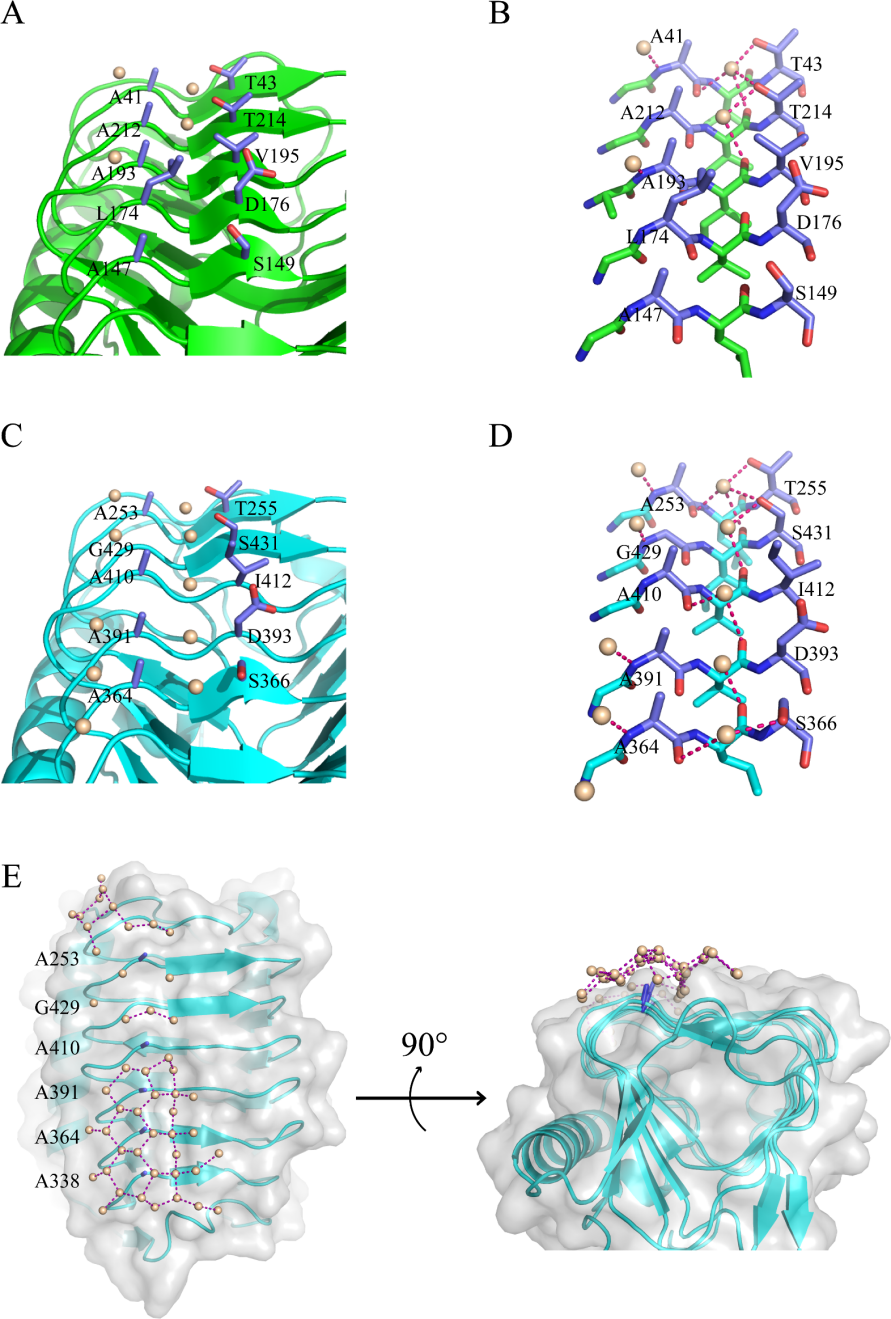
Predicted ice binding sites. (A) The predicted IBS on domain A. The two rows of aligned side chains are in blue, water molecules in gray. (B) Stick diagram of the IBS of domain A and surrounding water. Each water molecule formed at least one hydrogen bond with protein backbone. (C) The predicted IBS on domain B. The two rows of aligned side chains are in blue, water molecules in gray. One row of well packed water molecules was found between the two rows of aligned side chains. The other row of water is located on the other side of the aligned small side chains. (D) Stick diagram of the IBS of domain B and surrounding water. Each water molecule formed at least one hydrogen bond with protein backbone. (E) Water molecules form pentagonal and hexagonal rings at the IBS on domain B. Overall structure of domain B is displayed as cartoon (cyan). Ala row on the IBS of domain B is illustrated as stick diagram (blue).

Several studies have shown that IBS of AFPs are flat planes on the protein surfaces [23]. Sometimes the ice binding regions also consist of well aligned side chains and even well aligned water molecules [24]. In the IBPv structure, one flat plane was observed on the same surface for each domain as shown in Fig 1B and Fig 2, and they were not at the protein-protein contacts in the crystal. Two rows of linearly aligned side chains were found on each proposed IBS (Fig 4). The average distances between the neighboring Cα which these side chains attached to are 4.9 Å and 4.8 Å for domain A and B, respectively. The average distance between two neighboring water molecules is 4.8 Å on the proposed IBS of domain B. Since there are only three ordered water molecules on the proposed IBS of domain A, we could only calculate the nearest distance of two water molecules, which is 4.6 Å. Both the distances between water molecules and the distances between side chains match the 4.5Å repeats of water molecules on the basal and prism planes of hexagonal ice, and therefore, we propose this surface as the IBS for each domain.

To confirm the putative IBS, several mutations were generated to introduce a bulky side chain on the IBS (i.e., T214, A364, A391, A410, G429 and S431) in an attempt to disrupt its flatness and ice binding ability (Fig 4A & 4C). Our previous study showed that domain A did not significantly contribute to the overall TH of IBPv, whereas a recombinant protein of domain B (IBPv_b) exhibited comparable TH as the full length protein [47].

**Fig 5:**
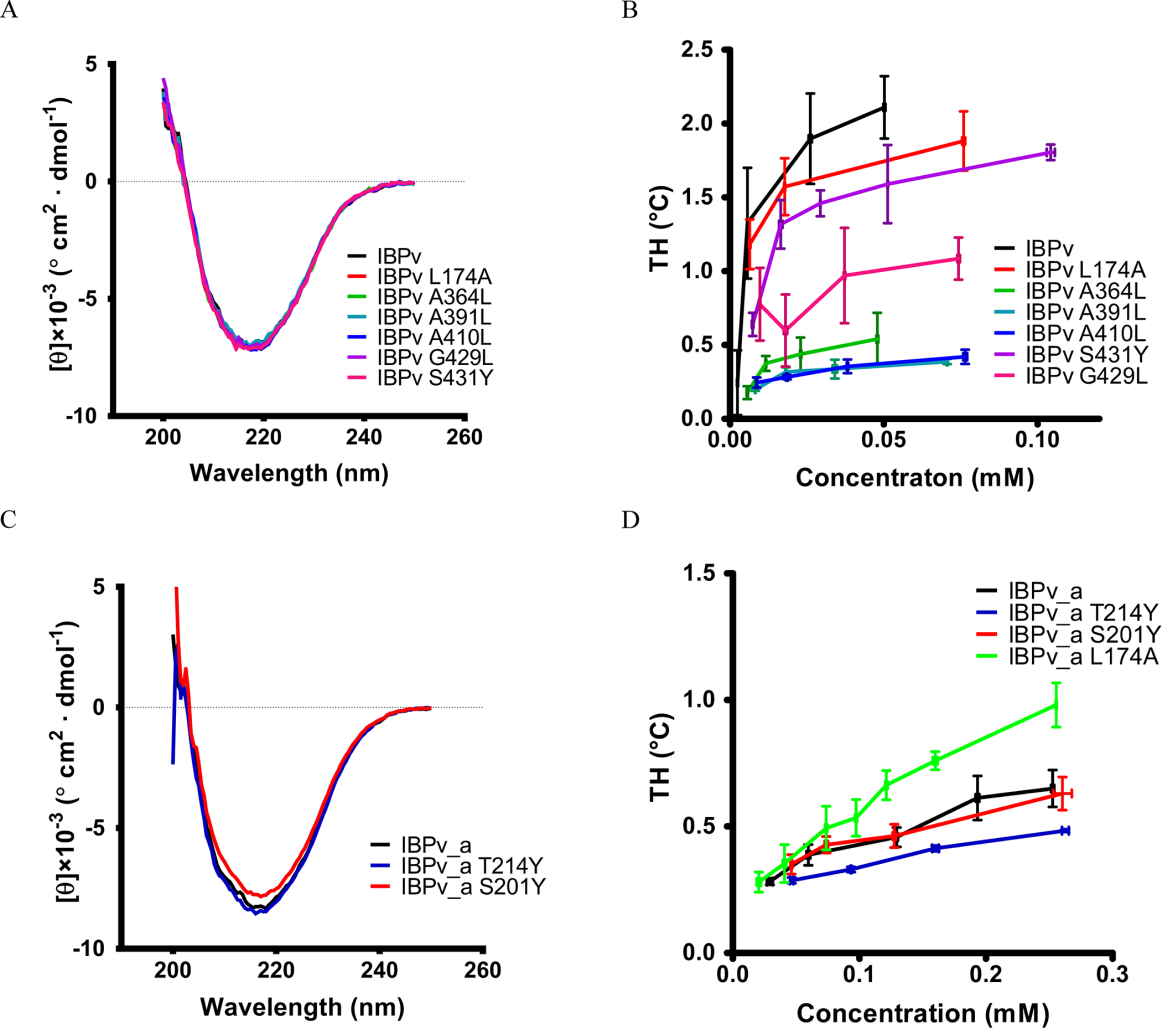
Antifreeze activities of the IBPv mutants. (A) The CD spectra of IBPv and its mutants. (B) TH of IBPv and its mutants. (C) The CD spectra of IBPv_a and its mutants. (D) TH of IBPv_a and its mutants.

Since domain B plays the predominant role in TH activity [47], mutations were introduced to the proposed IBS of domain B in the context of the full length protein IBPv. In the structure, the first row of the residues located between the two rows of well-aligned water molecules are all with small side chains (Ala or Gly) in domain B (Fig 4C & 4D). The residues on the other row are more varied, mainly containing Thr and Ser. Four mutations on the first row were generated: A364L, A391L, A410L and G429L. Our previous study showed that the circular dichroism (CD) spectrum of the full-length IBPv exhibited a single negative peak around 218 nm, suggesting that the predominant secondary structure was β sheet [47], which is consistent with the crystal structure. CD spectra of the recombinant mutants of IBPv were identical with the wild type protein, suggesting the point mutations did not alter the protein’s overall structure (Fig 5A). While the TH of the wild type IBPv is 2.11 ± 0.21°C at a protein concentration of 50 μM, the A364L, A391L, A410L and G429L mutants of IBPv exhibited drastically impaired TH, with values less than 1°C at a protein concentration of 50 μM. One amino acid on the second row of the residues, S431, was mutated to a Tyr. Compared to the wild type, the mutation did not have any effect on the CD spectra (Fig 5A), indicating no overall structural change. Although the effect of the S431Y mutation on the antifreeze activity of on IBPv was not comparable to A364L, A391L, A410L and G429L, it decreased the TH by 25% (Fig 5B). Hence, the mutagenesis results supported the hypothesis that these well aligned residues are important for antifreeze activity and confirmed the IBS of domain B.

In order to determine the proposed IBS of domain A by mutagenesis, the recombinant domain A (IBPv_a) was used instead of the full length protein, because domain B had much higher TH activity than domain A. Mutating one residue T214 at the proposed IBS and one residue away from the proposed IBS (S201) (S1 Fig) into Tyr had little effect on the CD spectra, suggesting the overall structure of the IBPv_a was unaltered (Fig 5C). The TH activity of the IBPv_a was 0.65 ± 0.07°C at a concentration of 0.25 mM (Fig 5D), which was consistent with previous observations [47]. However, changing the Thr residue at 214 into Tyr of IBPv_a decreased TH significantly (Fig 5D). As a negative control, the bulky residue was introduced at the loop region far away from the proposed IBS of IBPv_a (S201Y mutant), and this construct exhibited comparable TH to IBPv_a. These results supported the hypothesized region as the IBS of domain A.

### Structural comparison of the IBS from domain A and B

Domain A and B have 44% sequence identities and 61% positive matches, and the crystal structure showed that they both adopt a similar β helical structure (Fig 2B). Structural alignment of the backbones of these two domains exhibited a RMSD of 0.68Å, yet domain B clearly exhibits significantly higher ice-binding properties [47]. Interestingly, a layer of ordered water was observed on the IBS of domain B (Fig 4E), but not on domain A. These water molecules forms several pentagonal and hexagonal rings, which might serve as a linkage between the protein and ice.

Structural alignment at the IBS of both domains was used to elucidate the structural basis for the differences in ice binding activities. The first row of amino acids on the IBS reside on loops, and are Ala41-Ala212-Ala193-Leu174-Ala147 for domain A, and Ala253-Gly429-Ala410-Ala391-Ala364 for domain B (Fig 4). The second row of amino acids is part of the β-sheet structure on the IBS, and mainly consist of residues with moderately sized side chains (Thr and Ser) for both domains (Fig 4). All of the residues on the first row are small side chains except the residue Leu174. The isobutyl side chain of Leu174 points towards the second row of amino acids, which may block water molecules from binding to the groove between the two rows of side chain (Fig 4). We propose that this steric hindrance affects the binding affinity of domain A with ice, thus reducing the antifreeze activity.

Using the IBPv_a as a template, residue Leu174 was mutated to Ala. Comparing to the wild type IBPv_a, the mutant IBPv_a L174A significantly increased the TH activity, from 0.65 ± 0.07°C to 0.98 ± 0.08°C at a protein concentration of 0.25mM (Fig 5D). This result indicates that the side chain of the residue Leu affects antifreeze activity, and is probably due to direct interference with protein-ice binding. However, when the L174A mutation was introduced in the full length recombinant protein, TH did not increase (Fig 5B).

### Comparison of structurally similar AFPs

**Fig 6:**
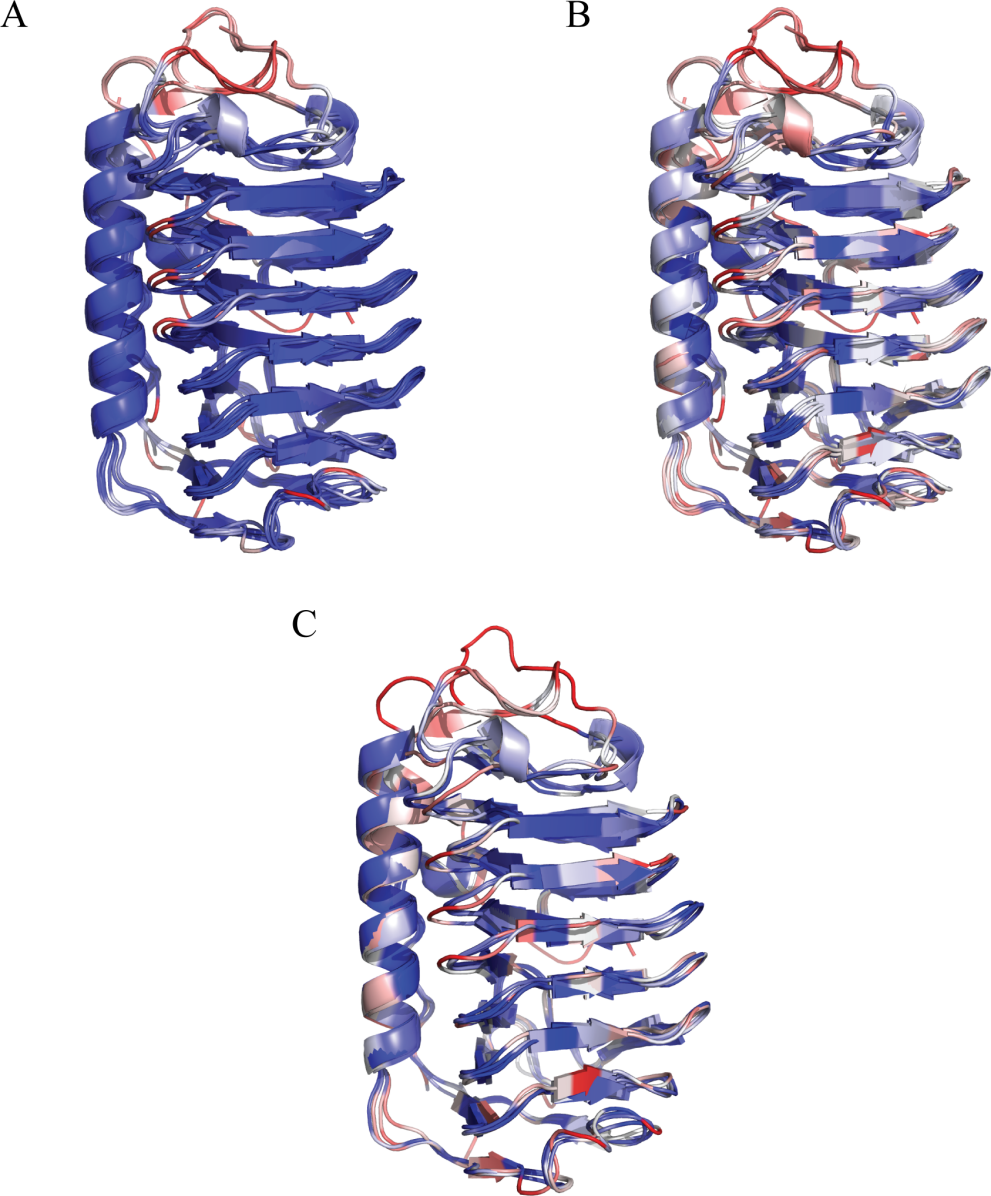
Structural comparison of homologous AFPs. (A) The structural alignment of IBPv_a, IBPv_b, *Col*AFP, *Le*IBP, *Ff*IBP, *Tis*AFP6 and *Tis*AFP8. Residues were colored by Qres, which was related to the distance of Cα after the alignment. Blue-white-red indicated from less deviance to large deviance between the Cα of aligned residues. (B) IBPv_a, IBPv_b, *Col*AFP, *Le*IBP, and *Tis*AFP6 were aligned by their structure data, but were colored according to primary sequence similarity using BLOSUM 60. Blue-white-red indicated from high similarity to low similarity. (C) The structural alignment of the hyperactive β helical AFPs. *Col*AFP, *Ff*IBP, *Tis*AFP8 and IBPv_b. Residues were colored by Qres values. Blue-white-red indicated from less deviance to large deviance between the Cα of aligned residues.

To date, several other AFPs that have been determined adopted the similar irregular β helical structure, namely *Le*IBP (from fungus *Leucosporidium sp*. *ay30*) [31], *Tis*AFP6 (from fungus *Typhula ishikariensis*) [30], *Tis*AFP8 (from fungus *Typhula ishikariensis*) [34], *Ff*IBP (from bacterium *Flavobacterium frigoris*) [33] and *Col*AFP (from bacterium *Colwellia sp*. *slw05*) [32]. The structure of IBPv_a, IBPv_b, *Le*IBP, *Tis*AFP6, *Tis*AFP8, *Ff*IBP and *Col*AFP were structurally aligned using MultiSeq [54], as shown in Fig 6. All these proteins shared similar structure with RMSD not exceeding 1.5Å for any two pairs. All protein structures in Fig 6A were colored according to their structural similarity (Qres in Multiseq) [54]. Blue-white-red indicated from less deviance to large deviance between the Cα of aligned residues. The structures were highly conserved on the β helix and α helix, with only minor differences observed on the loop regions. In addition, all residues were colored using BLOSUM 60 matrix in Fig 6B. Blue-white-red indicates from high to low sequence conservation. The sequence conserved region of these AFPs are the β helix hydrophobic core, the interface between α helix and β helix, and the turning regions of the β helix. The sequences at the IBS highly diverge among the aligned proteins. *Col*AFP [32], *Ff*IBP [33], *Tis*AFP8 [34] and IBPv_b [47] are categorized as hyperactive AFP. When only these 4 proteins were aligned, the sequences at the IBS still remained highly diverse (Fig 6C).

## Discussion

In this study, we determined the crystal structure of IBPv, which contains two homologous ice binding domains. This is the first reported crystal structure of a multi-domain AFP. Structural alignment indicates that the domains are structurally similar, with RMSD of 0.68Å on the protein backbone alignment. Each ice binding domain contains an irregular β-helix with a triangular cross-section, and a long α-helix running parallel along one side of the β-helix. A large number of hydrophobic residues were found inside the β-helix and at the interface between the α-helix and β-helix for each domain. One intra-domain disulfide bond was found in each domain as well. These results indicated that hydrophobic interactions and intra-domain disulfide bonds are the major forces that contribute to the overall stability of the protein, which may explain its thermal stability with a denaturation temperature of 53.5°C [47].

AFPs with similar β helical structure were aligned. Despite their high structural similarities, the sequence conservation is relatively low. These β helical AFPs exhibit high sequence conservation on the β helix core, the interface between α helix and β helix, and some turns on the β helix, which all are pivotal in maintaining the overall β helical protein structure. These findings suggested that β helical structure might have its advantageous in supporting the ice binding activity. One major advantage for AFPs to adopt β helical structure is that each coil in the solenoid has an axial rise of about 4.8Å [56], which perfectly matches the 4.5Å repeat of water molecules on both the basal and the prism planes of hexagonal ice.

AFPs bind specifically, rapidly and irreversibly to ice in aqueous solution [10, 57]. Different mechanisms of AFP binding to ice have been recently proposed [23]. Early studies on type I AFPs revealed a regular array of threonine side chains on the IBS, leading to the hypothesis that AFP binds with ice crystals through hydrogen bonds formed between water molecules and hydrophilic side chains [19, 58]. However, a number of studies have shown that the hydroxyl group on the side chains of AFPs are not required for the interaction with ice [39, 42, 59, 60]. Several AFP crystal structures have also shown that the IBSs consist mainly of hydrophobic residues [30, 31, 33, 34]. Cheng *et al* recently reported a hydrophilic residue, Ser, on the IBS of *Tis*AFP6 that might partially account for its inferior antifreeze activity compared to its isoform *Tis*AFP8 [34]. Therefore, these studies imply that hydrophobic side chains with flat-binding surface are vital for ice binding. It was proposed that ordered waters originally bound on the IBS might be released upon AFP adsorption to ice, and thus, the entropy increase drives the ice binding process [8, 42]. More recently, computer modelling studies have concluded that the IBS might organize water molecules into an ice-like pattern, and these ice-like liquid layers of water could then merge to the ice surface [43–45, 61]. This mechanism, called the ‘anchored clathrate water binding mechanism’, is supported by previous observations [20, 24]. In the crystal structure of IBPv, a layer of ordered water molecules forms a water network that consists of several pentagonal and hexagonal ring (Fig 4E) on the IBS of domain B. However, no such water network is observed on the IBS of domain A, which is the weaker one in ice binding activity of the two domains, suggesting that the presence of water network is correlated with the ice binding activity and thus supporting the anchored clathrate water binding mechanism.

Site-directed mutagenesis confirmed that the proposed ice binding site for each domain is located at the same region on the β-helix for IBPv. Many well aligned water molecules that are hydrogen bonding with the protein backbone were observed on the IBS for each domain. More ordered water molecules were found on the IBS of domain B, consistent with the previous observation that domain B has a much higher antifreeze activity [47]. The results suggest that the ice binding affinity of AFPs may be correlated to the number of ordered water molecules hydrogen-bonded to the IBS. These findings supported the anchored clathrate water binding mechanism, which proposed that AFPs do not directly bind with ice surface, but interact with ice through the ordered water molecules on their IBS instead.

Each domain of IBPv has two rows of water molecules on the IBS that form hydrogen bonds with the protein backbone, and more water molecules are found in the IBS of domain B compared to domain A (Fig 4B & 4D). The first row of water forms hydrogen bond with the amines of protein backbone. The second row of water forms a primary hydrogen bond with the carbonyl groups on the protein backbone, and some of those water molecules might even form a secondary hydrogen bond with the hydroxyl group on the side chains. In addition, the anchored water on the IBS of domain B are able to form several pentagonal and hexagonal rings. These anchored water molecules on the protein surface could further interact with water on the ice surface, since their spacing matched that of the basal and prism planes of hexagonal ice. These findings suggest that water-backbone interactions have important roles in allowing IBPv to bind with ice.

Two rows of side chains are well aligned with the two rows of water molecules on the IBS of each domain. The first row of the side chains are located between two rows of water molecules and are mainly small side chains except Leu 174 in domain A. The residues on the second row are mainly hydrophilic residues with hydroxyl groups that may potentially form hydrogen bonds. Close molecular comparison of the two domains revealed that the bulky side chain of Leu 174 on the IBS of domain A may account for its inferior antifreeze activity. The mutant L174A, in which the isobutyl group was replaced by a much smaller methyl group, enhanced the TH of IBPv_a, confirming this hypothesis. In contrast, mutating other small residues on the IBS of domain B into more bulky Leu significantly reduced the TH of IBPv. Therefore, the first row of the small, hydrophobic side chain residues play important roles in allowing two rows of water molecules to form hydrogen bonding interactions with the protein backbone. These findings suggest that the non-hindering effect at the IBS is vital for the ice binding of AFPs.

Although the L174A mutation produced a TH for domain A that was comparable to domain B, it did not increase, and even slightly impaired, the TH of the full length IBPv. Our previous work showed that domain B contributed the majority portion of the TH of IBPv [47]. Thus a relatively small increment of the TH in the domain A might not produce a noticeable effect on the TH of the full length protein. We also showed that IBPv_a and IBPv_b could work collaboratively to achieve a higher TH by preferentially targeting different ice planes [47]. We proposed that IBPv_a might preferentially target the prism planes of ice crystal, while IBPv_b might target the basal planes with higher affinity. The L174A mutation on IBPv_a might increase the binding affinity of domain A towards the basal planes, and decrease its binding towards the prism planes. If both domain B and the mutated domain A preferred to bind to the basal planes, the collaboration between these two domains might be lost, and therefore, it would not be surprising to observe a small decrease on the overall TH. A multi-domain AFP that targets different ice planes may have the potential to further suppress the freezing point and help the organism to tolerate more extreme freezing conditions.

## Supporting information

S1 Fig The location of S201 residue on domain A. S201 in Domain A is distal to the predicted IBS.
(DOCX)

S1 Table The TH data. (DOCX)

S2 Table The CD data. Mean residue molar ellipticity are displayed in 10^3^ deg cm^2^ · dmol^-1^.
(DOCX)

## Abbreviations

AFP: antifreeze protein
CD: circular dichroism
IBP: ice binding protein
IBPv: IBP from a Vostok Ice Core bacterial isolate
IBPv_a: A domain only IBPv
IBPv_b: B domain only IBPv
IBS: ice binding site
RI: recrystallization inhibition
TH: thermal hysteresis
