# Supplementary figures and images for: Structural basis of antifreeze activity of a bacterial multi-domain antifreeze protein
Source: PLoS One. 2017 Nov 6;12(11):e0187169. doi: 10.1371/journal.pone.0187169 (PMC5673226; doi:10.1371/journal.pone.0187169)

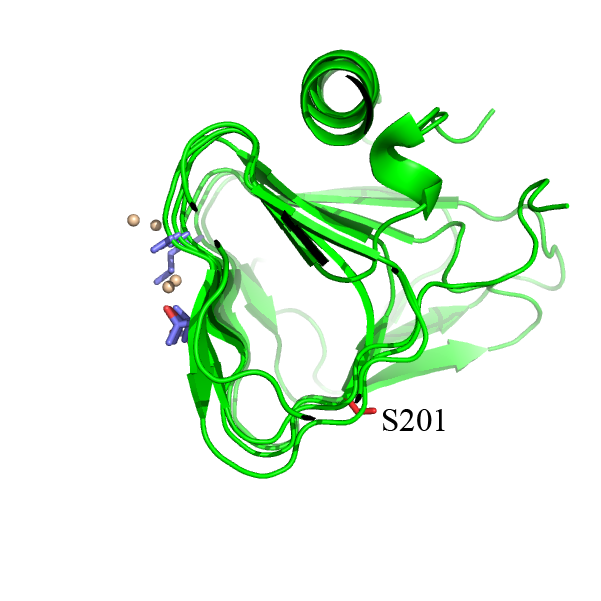


**S1 Fig. The location of S201 residue on domain A**. S201 in Domain A is distal to the predicted IBS.

Supplement: S1 Fig — S201 in Domain A is distal to the predicted IBS. (DOCX) [file pone.0187169.s001.docx]
