# Supplementary material for: Structural basis of antifreeze activity of a bacterial multi-domain antifreeze protein
Source: PLoS One. 2017 Nov 6;12(11):e0187169. doi: 10.1371/journal.pone.0187169 (PMC5673226; doi:10.1371/journal.pone.0187169)
Supplement: S1 Table — (DOCX) [file pone.0187169.s002.docx]

**S1 Table.** TH data

| IBPv | | | | | |  | IBPv G429L | | | | | |
| --- | --- | --- | --- | --- | --- | --- | --- | --- | --- | --- | --- | --- |
| Conc. (mM) | TH (°C) | | | | |  | Conc. (mM) | TH (°C) | | | | |
| 0.050 | 2.40 | 2.11 | 1.90 | 2.03 |  |  | 0.074 | 1.25 | 1.05 | 0.91 | 1.13 |  |
| 0.026 | 2.26 | 1.61 | 2.04 | 1.68 |  |  | 0.037 | 0.98 | 0.84 | 1.41 | 0.65 |  |
| 0.006 | 1.17 | 1.79 | 0.91 | 1.43 |  |  | 0.018 | 0.43 | 1.02 | 0.59 | 0.51 | 0.44 |
| 0.002 | 0.34 | 0.51 | 0.06 | 0.07 |  |  |  |  |  |  |  |  |
|  |  |  |  |  |  |  | IBPv_a | | | | | |
| IBPv L174A | | | | | |  | Conc. (mM) | TH (°C) | | | | |
| Conc. (mM) | TH (°C) | | | | |  | 0.253 | 0.74 | 0.57 | 0.62 | 0.67 |  |
| 0.076 | 1.81 | 1.64 | 2.08 | 2.10 | 1.78 |  | 0.193 | 0.57 | 0.68 | 0.51 | 0.69 |  |
| 0.018 | 1.42 | 1.39 | 1.53 | 1.66 | 1.86 |  | 0.129 | 0.44 | 0.42 | 0.51 | 0.46 |  |
| 0.006 | 1.27 | 1.00 | 1.37 | 1.09 |  |  | 0.060 | 0.40 | 0.44 | 0.36 | 0.35 |  |
|  |  |  |  |  |  |  | 0.029 | 0.28 | 0.30 | 0.27 | 0.28 |  |
| IBPv A364L | | | | | |  |  |  |  |  |  |  |
| Conc. (mM) | TH (°C) | | | | |  | IBPv_a L174A | | | | | |
| 0.048 | 0.73 | 0.34 | 0.38 | 0.70 | 0.55 |  | Conc. (mM) | TH (°C) | | | | |
| 0.023 | 0.54 | 0.38 | 0.42 | 0.29 | 0.56 |  | 0.256 | 0.92 | 0.94 | 1.08 |  |  |
| 0.012 | 0.30 | 0.41 | 0.40 | 0.39 |  |  | 0.160 | 0.77 | 0.72 | 0.79 |  |  |
| 0.006 | 0.16 | 0.17 | 0.24 | 0.14 |  |  | 0.121 | 0.73 | 0.63 | 0.63 |  |  |
|  |  |  |  |  |  |  | 0.097 | 0.45 | 0.53 | 0.52 | 0.52 | 0.65 |
| IBPv A391L | | | | | |  | 0.074 | 0.51 | 0.40 | 0.57 |  |  |
| Conc. (mM) | TH (°C) | | | | |  | 0.041 | 0.31 | 0.44 | 0.31 |  |  |
| 0.071 | 0.39 | 0.38 | 0.37 | 0.41 |  |  | 0.021 | 0.24 | 0.32 | 0.28 |  |  |
| 0.034 | 0.31 | 0.41 | 0.29 |  |  |  |  |  |  |  |  |  |
| 0.018 | 0.33 | 0.34 | 0.28 |  |  |  | IBPv_a T214Y | | | | | |
| 0.008 | 0.19 | 0.20 | 0.21 |  |  |  | Conc. (mM) | TH (°C) | | | | |
|  |  |  |  |  |  |  | 0.263 | 0.48 | 0.48 | 0.49 |  |  |
| IBPv A410L | | | | | |  | 0.160 | 0.40 | 0.42 | 0.42 |  |  |
| Conc. (mM) | TH (°C) | | | | |  | 0.093 | 0.33 | 0.32 | 0.34 |  |  |
| 0.076 | 0.40 | 0.38 | 0.41 | 0.49 |  |  | 0.047 | 0.28 | 0.28 | 0.30 |  |  |
| 0.038 | 0.34 | 0.30 | 0.37 | 0.41 |  |  |  |  |  |  |  |  |
| 0.019 | 0.30 | 0.29 | 0.27 | 0.27 |  |  | IBPv_a S201Y | | | | | |
| 0.009 | 0.28 | 0.22 | 0.21 | 0.27 |  |  | Conc. (mM) | TH (°C) | | | | |
|  |  |  |  |  |  |  | 0.260 | 0.56 | 0.64 | 0.69 |  |  |
| IBPv S431Y | | | | | |  | 0.128 | 0.44 | 0.53 | 0.43 | 0.45 |  |
| Conc. (mM) | TH (°C) | | | | |  | 0.074 | 0.45 | 0.43 | 0.38 | 0.45 |  |
| 0.104 | 1.80 | 1.78 | 1.76 | 1.88 |  |  | 0.046 | 0.40 | 0.32 | 0.36 | 0.32 |  |
| 0.051 | 1.43 | 1.57 | 1.97 | 1.39 |  |  |  |  |  |  |  |  |
| 0.029 | 1.39 | 1.40 | 1.58 | 1.47 |  |  |  |  |  |  |  |  |
| 0.016 | 1.08 | 1.36 | 1.37 | 1.46 |  |  |  |  |  |  |  |  |
| 0.007 | 0.59 | 0.73 | 0.68 | 0.56 |  |  |  |  |  |  |  |  |
