# Supplementary material for: Structural basis of antifreeze activity of a bacterial multi-domain antifreeze protein
Source: PLoS One. 2017 Nov 6;12(11):e0187169. doi: 10.1371/journal.pone.0187169 (PMC5673226; doi:10.1371/journal.pone.0187169)
Supplement: S2 Table — Mean residue molar ellipticity are displayed in 103 deg cm2 · dmol-1. (DOCX) [file pone.0187169.s003.docx]

**S2 Table.** CD data. Mean residue molar ellipticity are displayed in 10^3^ deg cm^2^ · dmol^-1^.

| Wavelength (nm) | IBPv | IBPv L174A | IBPv A364L | IBPv A391L | IBPv A410L |
| --- | --- | --- | --- | --- | --- |
| 250.0 | -0.09 | -0.14 | -0.12 | -0.12 | -0.05 |
| 249.5 | -0.07 | -0.08 | -0.09 | -0.07 | -0.05 |
| 249.0 | -0.07 | -0.07 | -0.09 | -0.10 | -0.05 |
| 248.5 | -0.06 | -0.08 | -0.07 | -0.10 | -0.06 |
| 248.0 | -0.07 | -0.09 | -0.07 | -0.09 | -0.11 |
| 247.5 | -0.04 | -0.06 | -0.07 | -0.13 | -0.04 |
| 247.0 | -0.06 | -0.03 | -0.02 | -0.10 | -0.01 |
| 246.5 | -0.05 | -0.08 | -0.06 | -0.05 | -0.10 |
| 246.0 | -0.13 | -0.15 | -0.12 | -0.10 | -0.15 |
| 245.5 | -0.19 | -0.15 | -0.16 | -0.10 | -0.17 |
| 245.0 | -0.18 | -0.17 | -0.21 | -0.18 | -0.20 |
| 244.5 | -0.12 | -0.12 | -0.14 | -0.11 | -0.19 |
| 244.0 | -0.19 | -0.14 | -0.16 | -0.13 | -0.17 |
| 243.5 | -0.26 | -0.23 | -0.17 | -0.14 | -0.19 |
| 243.0 | -0.31 | -0.30 | -0.21 | -0.18 | -0.27 |
| 242.5 | -0.31 | -0.39 | -0.26 | -0.28 | -0.36 |
| 242.0 | -0.31 | -0.42 | -0.35 | -0.35 | -0.39 |
| 241.5 | -0.36 | -0.40 | -0.34 | -0.36 | -0.38 |
| 241.0 | -0.39 | -0.40 | -0.29 | -0.40 | -0.39 |
| 240.5 | -0.40 | -0.41 | -0.40 | -0.45 | -0.45 |
| 240.0 | -0.46 | -0.49 | -0.45 | -0.48 | -0.51 |
| 239.5 | -0.53 | -0.55 | -0.52 | -0.55 | -0.53 |
| 239.0 | -0.60 | -0.63 | -0.64 | -0.62 | -0.63 |
| 238.5 | -0.66 | -0.69 | -0.68 | -0.60 | -0.65 |
| 238.0 | -0.72 | -0.78 | -0.69 | -0.65 | -0.69 |
| 237.5 | -0.87 | -0.82 | -0.81 | -0.78 | -0.81 |
| 237.0 | -0.96 | -0.87 | -0.87 | -0.83 | -0.92 |
| 236.5 | -0.99 | -0.96 | -0.94 | -0.90 | -0.96 |
| 236.0 | -1.11 | -1.10 | -1.10 | -0.99 | -1.05 |
| 235.5 | -1.11 | -1.15 | -1.25 | -1.09 | -1.17 |
| 235.0 | -1.26 | -1.27 | -1.32 | -1.25 | -1.33 |
| 234.5 | -1.46 | -1.49 | -1.43 | -1.44 | -1.50 |
| 234.0 | -1.64 | -1.67 | -1.66 | -1.57 | -1.68 |
| 233.5 | -1.85 | -1.85 | -1.94 | -1.80 | -1.84 |
| 233.0 | -2.02 | -2.03 | -2.09 | -1.93 | -2.07 |
| 232.5 | -2.22 | -2.26 | -2.28 | -2.20 | -2.30 |
| 232.0 | -2.35 | -2.39 | -2.41 | -2.36 | -2.38 |
| 231.5 | -2.51 | -2.60 | -2.56 | -2.52 | -2.59 |
| 231.0 | -2.83 | -2.91 | -2.86 | -2.88 | -2.90 |
| 230.5 | -3.10 | -3.06 | -3.16 | -3.11 | -3.09 |
| 230.0 | -3.32 | -3.35 | -3.50 | -3.27 | -3.41 |
| 229.5 | -3.65 | -3.70 | -3.77 | -3.53 | -3.66 |
| 229.0 | -3.84 | -3.89 | -3.86 | -3.80 | -3.85 |
| 228.5 | -4.11 | -4.19 | -4.15 | -4.05 | -4.16 |
| 228.0 | -4.31 | -4.44 | -4.35 | -4.23 | -4.36 |
| 227.5 | -4.53 | -4.54 | -4.56 | -4.44 | -4.59 |
| 227.0 | -4.72 | -4.87 | -4.85 | -4.65 | -4.88 |
| 226.5 | -5.02 | -5.17 | -5.07 | -4.95 | -5.10 |
| 226.0 | -5.15 | -5.22 | -5.22 | -5.07 | -5.23 |
| 225.5 | -5.36 | -5.39 | -5.42 | -5.17 | -5.41 |
| 225.0 | -5.60 | -5.59 | -5.67 | -5.54 | -5.67 |
| 224.5 | -5.73 | -5.73 | -5.83 | -5.65 | -5.84 |
| 224.0 | -5.89 | -5.92 | -5.95 | -5.82 | -6.04 |
| 223.5 | -5.96 | -5.99 | -6.08 | -6.04 | -6.12 |
| 223.0 | -6.09 | -6.13 | -6.20 | -6.08 | -6.24 |
| 222.5 | -6.29 | -6.35 | -6.33 | -6.19 | -6.42 |
| 222.0 | -6.42 | -6.48 | -6.52 | -6.42 | -6.55 |
| 221.5 | -6.48 | -6.64 | -6.62 | -6.45 | -6.72 |
| 221.0 | -6.65 | -6.65 | -6.87 | -6.50 | -6.93 |
| 220.5 | -6.78 | -6.74 | -6.97 | -6.68 | -6.99 |
| 220.0 | -6.83 | -6.90 | -6.99 | -6.87 | -7.07 |
| 219.5 | -6.83 | -7.00 | -7.05 | -6.90 | -7.15 |
| 219.0 | -6.87 | -7.01 | -7.03 | -6.78 | -7.10 |
| 218.5 | -6.92 | -6.92 | -7.07 | -6.79 | -7.17 |
| 218.0 | -6.93 | -6.89 | -7.15 | -6.87 | -7.14 |
| 217.5 | -7.09 | -6.97 | -7.09 | -6.96 | -7.18 |
| 217.0 | -7.05 | -7.03 | -7.12 | -6.99 | -7.13 |
| 216.5 | -6.95 | -6.87 | -7.05 | -6.83 | -6.98 |
| 216.0 | -6.89 | -6.93 | -7.01 | -6.85 | -7.04 |
| 215.5 | -6.75 | -6.90 | -6.94 | -6.84 | -6.94 |
| 215.0 | -6.71 | -6.81 | -6.80 | -6.64 | -6.79 |
| 214.5 | -6.79 | -6.91 | -6.97 | -6.66 | -6.99 |
| 214.0 | -6.54 | -6.85 | -6.75 | -6.56 | -6.85 |
| 213.5 | -6.35 | -6.69 | -6.65 | -6.34 | -6.59 |
| 213.0 | -6.35 | -6.57 | -6.52 | -6.28 | -6.52 |
| 212.5 | -6.12 | -6.18 | -6.42 | -6.07 | -6.35 |
| 212.0 | -6.04 | -6.10 | -6.26 | -6.02 | -6.27 |
| 211.5 | -5.82 | -5.95 | -6.11 | -5.78 | -6.16 |
| 211.0 | -5.43 | -5.62 | -5.86 | -5.52 | -5.82 |
| 210.5 | -5.26 | -5.45 | -5.56 | -5.49 | -5.54 |
| 210.0 | -5.14 | -5.37 | -5.32 | -5.29 | -5.37 |
| 209.5 | -4.86 | -5.18 | -5.21 | -5.00 | -5.17 |
| 209.0 | -4.68 | -4.85 | -5.05 | -4.69 | -4.87 |
| 208.5 | -4.38 | -4.66 | -4.54 | -4.26 | -4.56 |
| 208.0 | -4.04 | -4.32 | -4.10 | -3.92 | -4.30 |
| 207.5 | -3.41 | -3.65 | -3.49 | -3.39 | -3.59 |
| 207.0 | -2.79 | -3.14 | -3.02 | -2.74 | -3.04 |
| 206.5 | -2.30 | -2.36 | -2.42 | -2.05 | -2.39 |
| 206.0 | -1.82 | -1.84 | -1.88 | -1.71 | -1.61 |
| 205.5 | -1.26 | -1.59 | -1.26 | -1.29 | -1.25 |
| 205.0 | -0.64 | -0.96 | -0.90 | -0.68 | -0.69 |
| 204.5 | 0.00 | -0.17 | -0.29 | -0.26 | -0.23 |
| 204.0 | 0.40 | 0.17 | 0.25 | -0.12 | 0.43 |
| 203.5 | 1.40 | 0.80 | 0.67 | 1.11 | 0.84 |
| 203.0 | 2.05 | 1.40 | 1.26 | 1.85 | 1.45 |
| 202.5 | 2.04 | 1.81 | 1.40 | 1.66 | 1.53 |
| 202.0 | 2.20 | 2.28 | 1.73 | 2.21 | 1.70 |
| 201.5 | 2.26 | 2.73 | 2.49 | 2.73 | 2.43 |
| 201.0 | 2.26 | 3.11 | 2.69 | 2.62 | 3.29 |
| 200.5 | 2.37 | 3.25 | 2.99 | 2.64 | 3.39 |
| 200.0 | 3.97 | 3.10 | 3.68 | 3.81 | 3.58 |

| Wavelength (nm) | IBPv G429L | IBPv S431Y | IBPv_a | IBPv_a S201Y | IBPv_a T214Y |
| --- | --- | --- | --- | --- | --- |
| 250.0 | -0.08 | -0.09 | -0.03 | -0.02 | -0.04 |
| 249.5 | -0.05 | -0.05 | -0.06 | -0.02 | -0.01 |
| 249.0 | -0.04 | -0.02 | -0.08 | -0.05 | -0.01 |
| 248.5 | -0.08 | -0.08 | -0.06 | -0.06 | -0.03 |
| 248.0 | -0.07 | -0.08 | -0.06 | -0.02 | -0.05 |
| 247.5 | -0.09 | -0.05 | -0.06 | -0.04 | -0.03 |
| 247.0 | -0.09 | -0.04 | -0.05 | -0.08 | -0.04 |
| 246.5 | -0.05 | -0.05 | -0.08 | -0.07 | -0.06 |
| 246.0 | -0.10 | -0.06 | -0.09 | -0.05 | -0.10 |
| 245.5 | -0.13 | -0.05 | -0.10 | -0.06 | -0.08 |
| 245.0 | -0.17 | -0.15 | -0.15 | -0.12 | -0.10 |
| 244.5 | -0.11 | -0.15 | -0.15 | -0.13 | -0.15 |
| 244.0 | -0.13 | -0.13 | -0.16 | -0.14 | -0.14 |
| 243.5 | -0.15 | -0.17 | -0.17 | -0.16 | -0.18 |
| 243.0 | -0.20 | -0.23 | -0.23 | -0.20 | -0.25 |
| 242.5 | -0.30 | -0.32 | -0.27 | -0.23 | -0.24 |
| 242.0 | -0.34 | -0.29 | -0.30 | -0.24 | -0.27 |
| 241.5 | -0.38 | -0.35 | -0.31 | -0.32 | -0.34 |
| 241.0 | -0.38 | -0.37 | -0.43 | -0.42 | -0.42 |
| 240.5 | -0.43 | -0.43 | -0.51 | -0.47 | -0.48 |
| 240.0 | -0.53 | -0.46 | -0.60 | -0.52 | -0.53 |
| 239.5 | -0.54 | -0.52 | -0.63 | -0.57 | -0.60 |
| 239.0 | -0.57 | -0.60 | -0.68 | -0.64 | -0.67 |
| 238.5 | -0.67 | -0.60 | -0.79 | -0.71 | -0.81 |
| 238.0 | -0.75 | -0.67 | -0.88 | -0.76 | -0.91 |
| 237.5 | -0.78 | -0.75 | -0.98 | -0.85 | -0.98 |
| 237.0 | -0.84 | -0.83 | -1.10 | -0.97 | -1.08 |
| 236.5 | -0.93 | -0.90 | -1.19 | -1.06 | -1.20 |
| 236.0 | -1.08 | -1.05 | -1.34 | -1.22 | -1.32 |
| 235.5 | -1.17 | -1.13 | -1.49 | -1.36 | -1.47 |
| 235.0 | -1.28 | -1.26 | -1.66 | -1.49 | -1.64 |
| 234.5 | -1.50 | -1.47 | -1.83 | -1.65 | -1.79 |
| 234.0 | -1.60 | -1.65 | -1.99 | -1.84 | -2.01 |
| 233.5 | -1.87 | -1.83 | -2.19 | -1.99 | -2.23 |
| 233.0 | -2.09 | -2.05 | -2.41 | -2.18 | -2.40 |
| 232.5 | -2.22 | -2.26 | -2.63 | -2.37 | -2.61 |
| 232.0 | -2.41 | -2.46 | -2.84 | -2.61 | -2.82 |
| 231.5 | -2.59 | -2.70 | -3.03 | -2.82 | -3.05 |
| 231.0 | -2.93 | -2.94 | -3.32 | -3.04 | -3.33 |
| 230.5 | -3.11 | -3.16 | -3.59 | -3.30 | -3.61 |
| 230.0 | -3.34 | -3.43 | -3.86 | -3.59 | -3.94 |
| 229.5 | -3.65 | -3.62 | -4.11 | -3.82 | -4.23 |
| 229.0 | -3.87 | -3.89 | -4.44 | -4.07 | -4.48 |
| 228.5 | -4.21 | -4.26 | -4.72 | -4.36 | -4.76 |
| 228.0 | -4.38 | -4.44 | -4.98 | -4.67 | -5.06 |
| 227.5 | -4.53 | -4.56 | -5.26 | -4.90 | -5.33 |
| 227.0 | -4.78 | -4.84 | -5.51 | -5.11 | -5.61 |
| 226.5 | -5.02 | -5.13 | -5.78 | -5.39 | -5.84 |
| 226.0 | -5.13 | -5.27 | -6.03 | -5.60 | -6.07 |
| 225.5 | -5.35 | -5.38 | -6.23 | -5.84 | -6.31 |
| 225.0 | -5.63 | -5.61 | -6.36 | -6.00 | -6.49 |
| 224.5 | -5.77 | -5.81 | -6.56 | -6.23 | -6.67 |
| 224.0 | -5.94 | -6.00 | -6.81 | -6.37 | -6.82 |
| 223.5 | -6.09 | -6.12 | -6.97 | -6.56 | -7.05 |
| 223.0 | -6.20 | -6.16 | -7.17 | -6.75 | -7.22 |
| 222.5 | -6.33 | -6.37 | -7.33 | -6.89 | -7.35 |
| 222.0 | -6.49 | -6.50 | -7.39 | -7.00 | -7.51 |
| 221.5 | -6.63 | -6.63 | -7.53 | -7.15 | -7.63 |
| 221.0 | -6.78 | -6.84 | -7.67 | -7.31 | -7.78 |
| 220.5 | -6.85 | -6.93 | -7.80 | -7.45 | -7.94 |
| 220.0 | -6.92 | -7.00 | -7.86 | -7.52 | -8.05 |
| 219.5 | -7.01 | -7.10 | -8.00 | -7.59 | -8.14 |
| 219.0 | -6.95 | -7.09 | -8.11 | -7.60 | -8.24 |
| 218.5 | -6.93 | -7.09 | -8.21 | -7.67 | -8.33 |
| 218.0 | -6.99 | -7.08 | -8.34 | -7.78 | -8.46 |
| 217.5 | -7.03 | -7.11 | -8.28 | -7.79 | -8.47 |
| 217.0 | -7.05 | -7.15 | -8.24 | -7.84 | -8.42 |
| 216.5 | -7.04 | -7.07 | -8.26 | -7.76 | -8.49 |
| 216.0 | -6.98 | -7.10 | -8.25 | -7.76 | -8.55 |
| 215.5 | -6.84 | -7.04 | -8.31 | -7.76 | -8.42 |
| 215.0 | -6.66 | -6.94 | -8.23 | -7.65 | -8.36 |
| 214.5 | -6.91 | -7.14 | -8.15 | -7.68 | -8.38 |
| 214.0 | -6.75 | -6.89 | -8.03 | -7.59 | -8.32 |
| 213.5 | -6.49 | -6.59 | -7.76 | -7.46 | -8.18 |
| 213.0 | -6.47 | -6.62 | -7.66 | -7.30 | -7.99 |
| 212.5 | -6.32 | -6.43 | -7.69 | -7.16 | -7.97 |
| 212.0 | -6.21 | -6.25 | -7.60 | -7.03 | -7.79 |
| 211.5 | -5.85 | -6.09 | -7.40 | -6.93 | -7.59 |
| 211.0 | -5.59 | -5.79 | -7.23 | -6.79 | -7.55 |
| 210.5 | -5.54 | -5.54 | -7.14 | -6.59 | -7.34 |
| 210.0 | -5.47 | -5.46 | -7.03 | -6.41 | -7.19 |
| 209.5 | -5.03 | -5.24 | -6.90 | -6.25 | -7.04 |
| 209.0 | -4.75 | -4.89 | -6.76 | -6.07 | -6.98 |
| 208.5 | -4.31 | -4.53 | -6.51 | -5.79 | -6.77 |
| 208.0 | -3.98 | -4.33 | -6.13 | -5.50 | -6.51 |
| 207.5 | -3.51 | -3.54 | -5.73 | -5.14 | -5.94 |
| 207.0 | -3.01 | -2.95 | -5.29 | -4.70 | -5.59 |
| 206.5 | -2.44 | -2.68 | -4.94 | -4.35 | -5.30 |
| 206.0 | -2.03 | -2.09 | -4.41 | -3.63 | -4.55 |
| 205.5 | -1.26 | -1.55 | -3.70 | -3.19 | -3.97 |
| 205.0 | -0.70 | -1.01 | -3.12 | -2.34 | -3.25 |
| 204.5 | -0.24 | -0.45 | -2.49 | -1.67 | -2.92 |
| 204.0 | 0.57 | 0.29 | -1.88 | -1.59 | -2.23 |
| 203.5 | 1.26 | 0.51 | -1.30 | -1.08 | -1.40 |
| 203.0 | 1.57 | 0.86 | -0.47 | 0.55 | -0.43 |
| 202.5 | 1.58 | 1.58 | 0.69 | 1.59 | 0.30 |
| 202.0 | 2.10 | 2.06 | 0.58 | 0.93 | 1.00 |
| 201.5 | 2.76 | 2.36 | 0.68 | 1.09 | 0.40 |
| 201.0 | 3.12 | 2.41 | 0.90 | 2.74 | 2.67 |
| 200.5 | 3.97 | 2.79 | 1.79 | 6.13 | 2.12 |
| 200.0 | 4.41 | 3.40 | 3.03 | 7.12 | -2.36 |
